# Advanced Impression Techniques for Complex Maxillofacial Tissue Defects

**DOI:** 10.7759/cureus.116905

**Published:** 2026-09-25

**Authors:** Sakshi Sinha, Anna S Babu, Krishnapriya V N, Manju V

**Affiliations:** 1 Prosthodontics, Amrita School of Dentistry, Kochi, IND

**Keywords:** dental impression techniques, digital dentistry, extra oral prosthetic rehabilitation, impression techniques, maxilla-facial prosthodontics, maxillofacial defects, maxillofacial prosthodontics, maxillofacial rehabilitation, prosthetic rehabilitation, prosthodontics and dental implantology

## Abstract

This study aims to evaluate the various traditional, digital, and hybrid impression workflows in maxillofacial prosthodontics. It addresses the research question of how modern digital advancements overcome the limitations in accuracy, material waste, and data storage that are inherent in conventional techniques for complex intraoral and extraoral defects. A comprehensive literature search was conducted across PubMed, Scopus, and Google Scholar. Ninety-three English-language articles evaluating impression workflows for extraoral and intraoral prostheses were identified and analyzed. While conventional materials remain highly accessible and clinically useful, digital workflows, utilizing intraoral scanning, three-dimensional photogrammetry, and computer-aided workflows, offer superior precision for complex defects, streamline data storage, and allow virtual modifications. For implant-supported prostheses, both workflows demonstrate comparable accuracy. Hybrid approaches combining both techniques maximize outcomes in highly challenging scenarios. Although digital technologies present higher initial infrastructure costs and require specialized training, upcoming advancements in artificial intelligence-assisted design are expected to further transform the field. Digital and hybrid workflows significantly advance maxillofacial prosthodontics by improving precision and data management over traditional methods. Clinicians must understand the distinct indications and limitations of both approaches to deliver individualized, efficient, and optimized rehabilitative care tailored to diverse patient needs.

## Introduction and background

Maxillofacial prosthodontics is a specialized branch of dentistry dedicated to restoring or replacing stomatognathic and craniofacial structures using fixed or removable prostheses [1]. It addresses functional and aesthetic challenges caused by congenital anomalies, trauma [2], disease, or surgical interventions, significantly improving patients' quality of life [3,4]. Taking precise impressions is a key part of the treatment plan because it makes sure that anatomical structures are replicated correctly, which leads to the best fit, function, and appearance of the prosthesis [5]. Various authors have proposed a plethora of techniques for a successful impression. This narrative review provides a comprehensive review of these methods. Conventionally, achieving predictable outcomes has relied on selecting materials tailored to complex defect morphologies [6]. Irreversible hydrocolloid (alginate) remains widely used for diagnostic casts and interim prostheses [7] due to its cost-effectiveness, ease of manipulation, and hydrophilicity [8-10]. For definitive rehabilitations, addition silicone (polyvinyl siloxane (PVS)) provides superior dimensional stability, tear resistance, and elastic recovery [9,11,12]. Similarly, polyether elastomers offer exceptional hydrophilicity and fine-detail capture, rendering them valuable for recording deep undercuts and moisture-prone periocular or perioral tissues [13,14]. However, conventional impression techniques often pose risks in complex defects, such as soft-tissue displacement, material locking in severe undercuts, and patient discomfort. While digital and hybrid workflows increasingly overcome these anatomical hurdles, a consolidated framework for their selection is lacking. Therefore, this review evaluates conventional impression techniques alongside contemporary digital and hybrid alternatives to guide clinical decision-making in complex maxillofacial rehabilitation.

## Review

Methodology

A comprehensive literature search was conducted across PubMed, Scopus, and Google Scholar to evaluate impression workflows in maxillofacial prosthodontics. The search focused on English-language articles detailing conventional, digital, and hybrid techniques for both intraoral and extraoral defects. A total of 93 eligible articles were identified and analyzed to synthesize clinical protocols, material selections, and technical outcomes across diverse anatomical presentations.

Impressions for ocular defects

Loss of an eye leads to the loss of vision and depth perception [15]. Surgical eye removal, including evisceration, enucleation, or exenteration [16], impacts socket health and prosthesis mobility. Proper socket impressions are critical for a good fit of the prosthesis [17]. Earlier techniques involved the use of alginate material to get a static shape of the eye socket (Table 1). There has been a shift towards the use of PVS and elastomers to capture the physiological movements of the eye socket. Newer techniques also focus on recording the final impressions using a special tray fabricated on the primary cast for better detail reproduction.

**Table 1:** Impression techniques for ocular defects. PMMA: polymethyl methacrylate; PVS: polyvinyl siloxane.

Authors	Tray/ material type	Key methodology
Allen and Webster [18]	Stock tray	Injected ophthalmic alginate; adjusted eyelids to match the healthy side
Chalian et al. [2]	Modified stock prosthesis	Trimmed and filled existing stock prostheses with material for a custom fit
Engelmier [19]	Ticonium tray	Introduced autoclavable metal trays for the procedure
Sykes et al. [20]	Plastic/rubber ball	Plastic molded over half a rubber ball using a hollow tube for injection
Taylor [4]	Perforated tray	Alginate injected via syringe followed by tray placement; boxed and poured
Kaur et al. [21]	Perforated PMMA tray	Adapted a stock eye with an orientation tube to capture socket and eyelid movement
Jethwani et al. [22]	Hollow-handled acrylic tray	Injected elastomer through the handle while capturing active eye movement
Tripuraneni et al. [23]	Heavy-body PVS	Direct socket injection; material sets while the eyelid remains closed
Bali et al. [24]	Wax and alginate	Softened wax for diagnostic impression; alginate injected and reinforced with plaster
Khungar et al. [25]	Putty and PVS	Putty-derived custom tray; PVS captured motion; wax pattern poured in two stages

Impressions for orbital defects

Orbital exenteration involves removing the eye and surrounding tissues, often for cancers like basal cell carcinoma, melanoma, or infections such as mucormycosis [26]. The extent varies with disease severity, from total to eyelid-sparing removal. Post-surgery, an orbital prosthesis, secured with adhesives, implants, or magnets, restores form and function by covering the defect. Accurate impressions and fitting are critical for optimal outcomes (Table 2). The shift toward using PVS in more recent techniques provides significantly greater dimensional stability compared to the hydrocolloids used in earlier facial moulage protocols.

**Table 2:** Impression techniques for orbital defects. VPS: vinyl polysiloxane.

Authors	Tray/material type	Key methodology
Brown [27]	Reversible hydrocolloid, wax collar, plaster	Supine; used a wax collar to confine the hydrocolloid; reinforced with quick-setting plaster
Levy et al. [28]	Alginate, gauze pads, plaster of Paris	Facial moulage method; used gauze to retain plaster and upright positioning to minimize gravity-induced tissue distortion
Thomas [29]	Alginate, adhesive foam tape, gauze, plaster	Masked forehead to lip with foam tape; lubricated edges and blocked nostrils; supported alginate with gauze/plaster
Karakoca et al. [30]	Light-body VPS, magnetic abutments	Implant-retained: acrylic substructure luted over a cast; injected VPS for soft tissue capture
Bindhoo and Aruna [31]	Irreversible hydrocolloid, nasal tubes	Upright; full facial impression (forehead to nose); utilized nasal tubes for breathing and gentle boxing to prevent tissue compression
Beumer et al. [1]	Polysulfide, adhesive-coated gauze, plaster	Applied a thin layer of polysulfide directly to the defect; reinforced with adhesive gauze and multiple plaster layers

Impressions for auricular defects

Auricular prostheses restore ears lost due to congenital defects, trauma, or surgery. Total loss allows easier replication; partial defects and microtia require complex designs [32,33]. Accurate restoration depends on preoperative impressions (Table 3), photos, and proper tissue bed preparation with grafts or flaps, preserving key landmarks like the tragus.

**Table 3:** Impression techniques for auricular defects. VPS: vinyl polysiloxane; PVS: polyvinyl siloxane.

Authors	Tray/material type	Key methodology
Brown [27]	Reversible hydrocolloid, wax collar	Patient positioned for alignment; wax collar used to confine the hydrocolloid for accuracy
Wolfaardt and Coss [3]	Resin substructure (bar/clip), custom tray, polyether	Used a custom tray to capture the defect with polyether over a bar-and-clip substructure
Cheng et al. [5]	Thermoformed shell, alginate	Thermoformed shell guided spatial relations; a sculpted wax ear was used to cast a bar-guiding shell
Wang [34]	CT-guided planning, face-bow, radiopaque resin	Used a face-bow for landmarks and radiopaque models for site identification
Mathews et al. [35]	Hydrocolloid, rigid tray, petroleum jelly	Rigid tray boxing with reference lines marked on the patient before material injection
Kubon and Anderson [36]	Resin-splinted copings, VPS, tray resin	Splinting protocol: copings splinted with resin bars to prevent movement; VPS used with tray resin under cooling
Jain et al. [37]	Acrylic substructure, PVS	Recorded mandibular movement during the impression to optimize bar and clip positioning
Mohamed et al. [38]	Alginate (triple-layer), custom tray	Used a specialized triple-layer injection technique for high-detail contour capture
Guttal et al. [39]	Light-body PVS, putty, impression compound	Abutments blocked with wax; light-body PVS reinforced with putty and compound
Waqas et al. [40]	Light-body and hard silicone	Two-step method; initial wash followed by a functional reline during tissue movement

Impressions for nasal defects

Nasal prostheses restore function and aesthetics after rhinectomy, improving patients’ psychological and social well-being. Permanent prostheses, crafted after tissue stabilization, use detailed impressions to capture defect anatomy accurately, often employing facial moulage and custom trays (Table 4) [41]. Elastic impression materials, such as hydrophilic PVS and polyether, with good flow are preferred, and blocking the nasal passage with gauze is essential to prevent material from entering during the impression [42].

**Table 4:** Impression techniques for nasal defects. RTV: room temperature vulcanizing; VPS: vinyl polysiloxane; PVS: polyvinyl siloxane; SS: stainless steel.

Authors	Tray/material type	Key methodology
Hutcheson and Udagama [42]	Silicone rubber, wire mesh, hydrocolloid	Upright position; reinforced silicone with wire mesh; used hydrocolloid for disfigured areas to create a diagnostic wax sculpting
Zaki and Myers [43]	RTV silicone, cotton applicators, spatula	Septal defect: injected silicone using cotton applicators as handles; adapted material with a spatula to capture one side at a time
Blind et al. [44]	Alginate, nasal tamponades	Performed under local anesthesia; used tamponades to block the cavity; removed the impression in two halves
Guttal et al. [39]	Medium-body VPS, custom tray, gauze	Nasal defect packed with moist gauze; impression posts used with a custom tray to fabricate the master cast
Beumer et al. [1]	Light-body rubber base, gauze, plaster	Applied elastomeric material directly to the defect; reinforced with adhesive-coated gauze and successive plaster layers
Shetty et al. [45]	Alginate, polyether, paper clips, plaster	Initial alginate reinforced with paper clips/plaster; final polyether impression taken during facial movements
Gupta et al. [46]	Hydrophilic VPS	Patient positioned semi-upright to minimize soft tissue distortion; used hydrophilic VPS for better moisture tolerance
Javanmard et al. [47]	Light/medium PVS	Pins splinted with acrylic for stability; PVS supported by plaster to create a high-accuracy Type IV die stone
Motsch et al. [48]	Wax model, facial impression	Captured anatomy before subtotal rhinectomy to ensure the final prosthesis replicated the original anatomy
Toumaj et al. [49]	Alginate, modified box, suction tube	Used a modified cardboard box as a tray and a suction tube to maintain the airway while gauze blocked the lesion

Impressions for maxillary defects

Patients with maxillary defects face challenges in mastication, speech, and swallowing, necessitating prostheses that restore normal physiological function [50]. Velopharyngeal deficits, arising from congenital or acquired causes, impair speech and swallowing by disrupting closure between the oral and nasal cavities [4,51]. Prosthetic obturators, tailored to defect type, restore separation of oral and nasal spaces, control nasal emission, and facilitate swallowing. Table 5 shows the various impression techniques to record the maxillary defects.

**Table 5:** Impression techniques for maxillary defects.

Authors	Clinical focus	Materials and tools	Key methodology
Adisman et al. [52]	Palatal defect	Light-body rubber base, syringe port	Seated a custom tray with regular-body material, then injected light-body through a port to capture undercuts
Bagis et al. [53]	Excess material control	Alginate, anesthetic-soaked tampon	Used a Vaseline-coated tampon with a retrieval string to block the defect and prevent material ingestion or flow
Anandakrishna [54]	Metal framework	Alginate, metal framework design	Surveyed a diagnostic cast to design a metal framework; secondary impressions were used for the final refractory cast
Varghese [55]	Soft palate/speech bulb	Putty, acrylic soft liner	Used head movements for border molding; the soft liner was refined during function and converted into a speech bulb
Buzayan [56]	Trismus	Sectioned stock and custom trays	Stock and custom trays were cut into segments to allow for separate right and left side impressions
Dudley et al. [57]	Palatal defect	CoeComfort, Monophase silicone	Recorded functional movements with tissue conditioner followed by a final silicone wash
Chandraker [58]	Large undercuts	Silicone putty, gauze	Putty packed incrementally with petroleum jelly; impression removed in pieces and reassembled for stability
Dutta et al. [59]	Trismus	Light-body silicone, green stick	Used custom trays with escape holes for light-body silicone; green stick compound shaped the bulb wall
Okazaki et al. [60]	Infant cleft palate	Silicone putty, 3D scanning	Compared conventional putty impressions/stone casts with intraoral scans and 3D-printed epoxy models

Impressions for mandibular defects

Advanced orofacial cancer cases need mandibulectomy and neck dissection. Reconstruction with flaps (e.g., pectoralis major) and osseointegrated implants restores form and function [61]. Prosthetic rehabilitation depends on tissue loss and dentition. Implant-supported options improve outcomes in larger defects. Success relies on precise surgical planning, prosthetic design, and follow-up [62]. Impression techniques to record the defect are mentioned in Table 6.

**Table 6:** Impression techniques for mandibular defects. ZOE: zinc oxide eugenol; PVS: polyvinyl siloxane.

Authors	Tray/material type	Key methodology
Cantor and Curtis [62]	Thin mix alginate, custom tray	Tray stabilized by vertical acrylic columns; captured the alveololingual sulcus during functional movements
Cheng et al. [5]	Sectional trays, cyanoacrylate	Used two separate trays to manage severe lingual undercuts; joined them with adhesive post-impression
Pekkan et al. [63]	Red/green modelling compound, ZOE paste	Neutral zone: molded compound using functional movements; followed by a final ZOE wash
Patil [64]	Compound, ZOE wash, sectional tray	Modified tray with compound for preliminary records; final wash in ZOE after sectional border molding
Aruna and Thulasingam [65]	SS wire, acrylic, impression compound	Two-part guiding flange used to guide the mandible into centric relation during the impression
Schneider et al. [66]	VARIOmulti abutments, PVS, resin	Impression copings stabilized with light-cured resin in a custom tray using PVS
Blanco and Ostrosky [67]	Taller copings, hydrocolloid, PVS	Controlled soft tissue overgrowth with tall copings; initial hydrocolloid tray followed by final PVS
Mundhe et al. [68]	Right/left sectional trays, putty silicone	Sectional trays stabilized intraorally with compound; removed as a single unit to ensure spatial accuracy
Jambhekar et al. [69]	Polyether, stent, open-tray	Emergence profile preserved with a stent; used open-tray polyether technique for final implant positioning
Tian et al. [61]	Closed and open-tray posts, PVS	Two-step protocol: Initial closed-tray followed by splinted open-tray posts for high-precision implant capture

Impressions for combination defects

Combination defects in maxillofacial prosthodontics involve the loss or malformation of multiple facial and cranial structures, such as the midface, nasal area, orbit, oral cavity, or cranial vault. Rehabilitation requires a multidisciplinary approach using advanced techniques, craniofacial implants, and digital planning to restore form and function. Midfacial defects demand precise impression techniques due to their complexity (Table 7) [70]. The primary difficulty in these cases is the orientation of the intraoral maxillary component to the extraoral facial component. The use of putty indices acts as a physical key to ensure these two distinct impressions align perfectly on the master cast [71].

**Table 7:** Impression techniques for combination defects. PVS: polyvinyl siloxane.

Authors	Tray/material type	Key methodology
Fernandes et al. [72]	Gauze, alginate, hydrocolloid, plaster	Packed nasal cavity with gauze; took a maxillary alginate impression followed by a facial hydrocolloid moulage reinforced with plaster
Fernandes et al. [72]	Wax, alginate, gauze, plaster	Used wax to create a box around the nose and lip for intranasal alginate; supported the material with embedded gauze and plaster
Veerareddy et al. [71]	Putty, cotton, alginate, plaster	Customized plastic basket used as an impression tray with putty indices; facial moulage with orthopedic plaster reinforced by "tripod mounds" for stability
Kurien et al. [73]	Syringe barrel tray, PVS (Putty/light body), alginate, plaster	Used a syringe barrel as a mini-tray for nasal PVS; followed by a midfacial alginate moulage reinforced with gauze and plaster
Ravi et al. [74]	Compound, silicone, alginate	For patients with restricted opening; each quadrant recorded separately with varying materials and reassembled post-retrieval

Cranial defects from trauma, surgery, or congenital issues affect appearance and brain protection, often causing neurological symptoms that improve with rehabilitation [75]. Kharade et al. [75] recorded cranial defects using irreversible hydrocolloid supported by layered dental plaster to avoid distortion and ensure an accurate moulage. Mathew et al. [76] applied petroleum jelly and plastic over hair and soft tissue before making an alginate impression, reinforcing it with cotton fibers and Type II plaster for rigidity and accuracy.

Impressions for extremity defects

Finger and partial-finger amputations, often caused by trauma or congenital issues, pose significant aesthetic and functional challenges. Prosthetic restoration, especially when multiple fingers are lost, is complex but essential for improving quality of life [77]. Impression techniques have traditionally utilized jars and containers as an impression tray to record a full hand impression of the defect as well as the unaffected side (Table 8).

**Table 8:** Impression techniques for extremity defects.

Authors	Tray/material type	Key methodology
Clarke et al. [78]	Latex, ice water	Hand placed in latex repeatedly to create a thick rubber mold
Pillet [79]	Alginate	Used a low-viscosity material to record the details in a long, cylindrical container
Tripathi et al. [80]	Addition silicone	Plastic impression caps were used with vent holes to make an impression using addition silicone
Garg et al. [77]	Irreversible hydrocolloid	Lubricated the hand with petroleum jelly before taking a hydrocolloid impression in a wide container
Dathan et al. [81]	Hydrocolloid, plaster	Painted hydrocolloid over the defect, which was covered by surgical gauze and dental plaster

Digital impressions

The traditional workflow for impression making in maxillofacial prosthetics typically involves a moulage, which uses physical materials like irreversible hydrocolloid (alginate) or silicone polymers to create an impression of the defect. While effective, this conventional approach is often limited by the weight of the impression material, which can displace soft tissues and lead to dimensional inaccuracies, particularly in large extraoral defects. Furthermore, the process can be traumatic or uncomfortable for patients, especially those with compromised airways or pediatric patients who struggle to remain still during the setting time of the material [82-84].

Digital technology has revolutionized prosthodontics by replacing error‑prone physical impressions with precise, non-contact three‑dimensional surface scans that improve accuracy, clinical efficiency, and patient comfort. The digital workflow for maxillofacial defect rehabilitation comprises four interconnected stages: visualization, design, manufacturing, and clinical evaluation, beginning with cross-sectional medical imaging (CT, CBCT, MRI) or optical surface scanning to produce polygonal surface meshes (e.g., STL, PLY, OBJ files) [85,86]. These multimodal datasets are merged in specialized CAD software, often employing digital mirroring of the healthy contralateral anatomy for unilateral defects, and subsequently transferred to CAM systems (e.g., stereolithography, selective laser sintering, or direct silicone additive manufacturing) for rapid prototyping and fabrication [87]. Standardized libraries of anatomical models (e.g., Ear&Nose Digital Library, edentulous obturator databases) further streamline the design phase when patient‑specific baseline data are unavailable [8]. Following fabrication, final prostheses are evaluated subjectively for aesthetic integration and objectively via computerized fit verification or digitized speech analysis. Incorporating 3D facial scanning and stereophotogrammetry allows for the precise co-registration of intraoral scan data and CBCT volumes to create a comprehensive "virtual patient," aiding complex smile design, soft-tissue tracking, and craniofacial surgical planning [88,89]. Although high equipment costs, learning curves, and software complexities have historically limited widespread adoption, modern structured‑light scanners and stereophotogrammetric systems routinely achieve submillimeter accuracy with minimal linear distortion, making digital workflows increasingly indispensable [90,91].

The data acquisition phase of this workflow utilizes various optical and radiographic modalities depending on the defect location and tissue stability. For extraoral prostheses like ears or noses, non-contact optical surface scanners and stereophotogrammetry systems are preferred as they capture intricate surface topography and dynamic margins without gravitational tissue displacement. For intraoral defects, such as those following a maxillectomy, intraoral scanners (IOS) provide a comfortable, contactless method for recording the remaining dentition and peripheral defect margins, though deep, light-inaccessible undercuts and moist tissues still require hybrid alignment with CBCT volumetric data for complete reconstruction. While digital optical scanners excel at capturing static anatomy, they cannot actively border-mold mobile muscular peripheries (e.g., soft palate seal in maxillectomy, dynamic eye-socket movements in orbital defects).

Once the digital impression is captured, CAD software such as Mimics (Materialise NV, Leuven, Belgium) or MeshMixer (Autodesk Inc., San Rafael, CA, USA) allows clinicians to segment anatomical structures, separate boundary lines, and perform virtual surgical resections or prosthetic planning [92,93]. This digital paradigm significantly reduces laboratory turnaround time, eliminates plaster cast storage, and enables the permanent archiving of digital master models, allowing for rapid, identical re-fabrication if a prosthesis is degraded, lost, or damaged. As advanced digital technologies and artificial intelligence continue to evolve, particularly through automated image segmentation, algorithmic anatomical feature matching, and neural network-driven digital shade determination, these workflows are becoming the standard for achieving high-fidelity aesthetic and functional outcomes in complex maxillofacial rehabilitation [87,94,95].

## Conclusions

The clinical management of maxillofacial defects remains an intricate challenge due to the heterogeneity of anatomical loss and unique physiological constraints. As evidenced by the evolution from plaster models to contemporary functional relining protocols, no singular gold standard applies universally. Practitioners must routinely adapt procedures to navigate complex undercuts, limited mouth opening, and fragile mucosal tissues. This necessity for customization underscores that every defect requires a bespoke combination of tray design, material selection, and patient positioning to achieve an accurate master cast. While traditional materials like irreversible hydrocolloids and PVS remain reliable cornerstones, the paradigm is shifting significantly toward digital technology. The integration of intraoral and extraoral scanning, CAD/CAM workflows, and 3D printing has revolutionized diagnostics and prosthetic design. This transition enhances anatomical precision while mitigating risks like material aspiration or soft-tissue distortion under material weight. Furthermore, digital workflows facilitate a more predictable, less invasive experience, particularly in pediatric cleft cases or extensive mid-facial reconstructions. By leveraging preoperative records and virtual planning, clinicians can replicate original anatomy with a level of fidelity unattainable through manual wax sculpting alone. Moving forward, the synergy between traditional expertise and emerging digital tools will define the next era of maxillofacial rehabilitation, ensuring optimal functional and aesthetic outcomes for the most complex defects.
